# Supplementary material for: Self-Assembled Metal–Organic Biohybrids (MOBs) Using Copper and Silver for Cell Studies
Source: Nanomaterials (Basel). 2019 Sep 8;9(9):1282. doi: 10.3390/nano9091282 (PMC6781094; doi:10.3390/nano9091282)
Supplement: Supplementary file 1 [file nanomaterials-09-01282-s001.pdf]

SUPPORTING MATERIALS

Self-assembled metal-organic biohybrids (MOBs) using copper and silver for cell studies

Neha Karekar[1+], Anik Karan[2+], Elnaz Khezerlou[2], Neela Prajapati[2], Chelsea D. Pernici[3], Teresa A. Murray[2], and Mark A. DeCoster [2,4,*]

[1] Cellular Neuroscience Laboratory, Molecular Science and Nanotechnology, Applied and Natural Sciences, Louisiana Tech University, Ruston, LA 71270, USA; nehak2712@gmail.com (N.K.)

[2] Cellular Neuroscience Laboratory, Biomedical Engineering, College of Engineering and Sciences, Louisiana Tech University, Ruston, LA 71270, USA; aka029@latech.edu (A.K.); ekh008@latech.edu (E.K.); npr008@latech.edu (N.P.); tmurray@latech.edu (T.M.)

[3] College of Pharmacy, University of Utah, Salt Lake City, UT 84112, USA; chelsea.pernici@utah.edu (C.P.)

[4] Cellular Neuroscience Laboratory, Institute for Micromanufacturing, College of Engineering and Sciences, Louisiana Tech University, Ruston, LA 71270, USA

[+] N. Karekar and A. Karan contributed equally to this work as co-first authors.

***** Correspondence: decoster@latech.edu (M.D.); Tel.: +1-318-257-5118; Fax: +1-318-257-4000

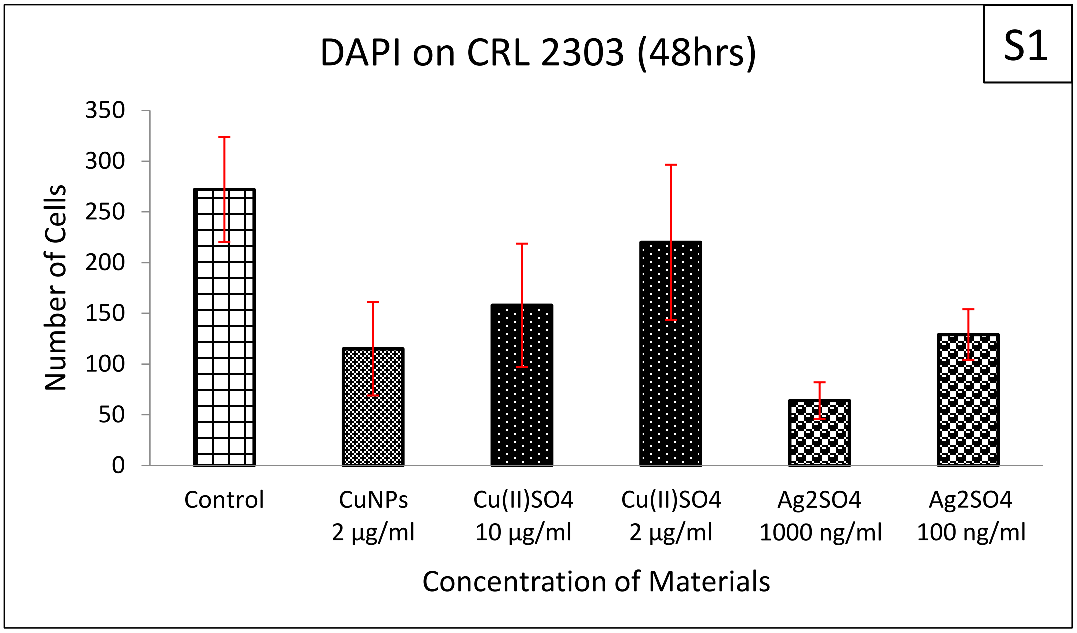
S1
DAPI on CRL 2303 (48hrs)
Number of Cells
350
300
250
200
150
100
50
0
Control
CuNPs 2 µg/ml
Cu(II)SO4 10 µg/ml
Cu(II)SO4 2 µg/ml
Ag2SO4 1000 ng/ml
Ag2SO4 100 ng/ml
Concentration of Materials

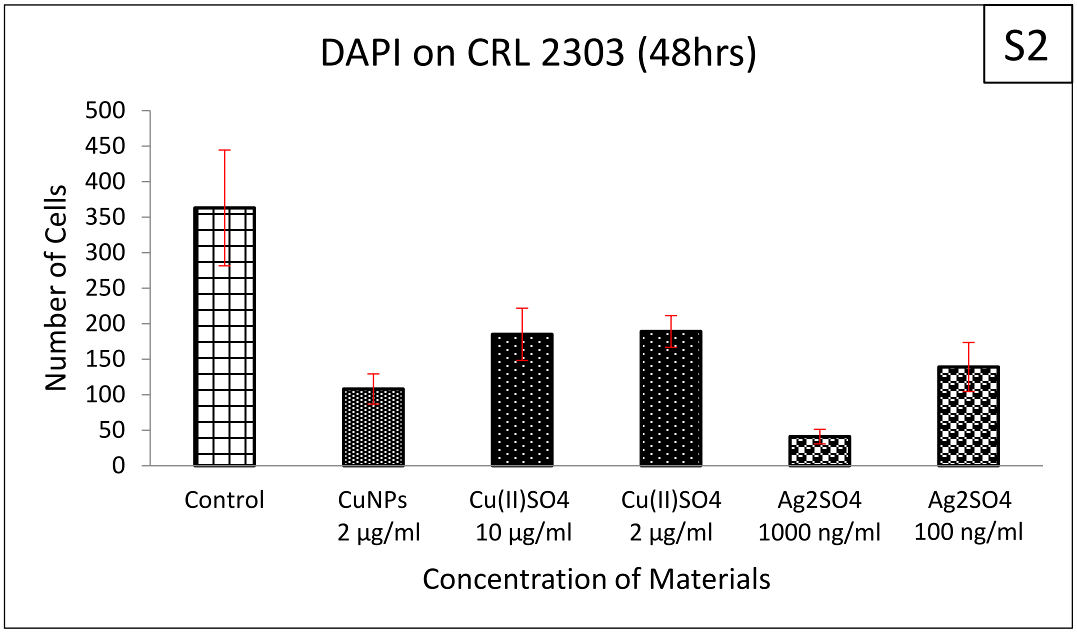
S2
DAPI on CRL 2303 (48hrs)
Number of Cells
500
450
400
350
300
250
200
150
100
50
0
Control
CuNPs 2 µg/ml
Cu(II)SO4 10 µg/ml
Cu(II)SO4 2 µg/ml
Ag2SO4 1000 ng/ml
Ag2SO4 100 ng/ml
Concentration of Materials

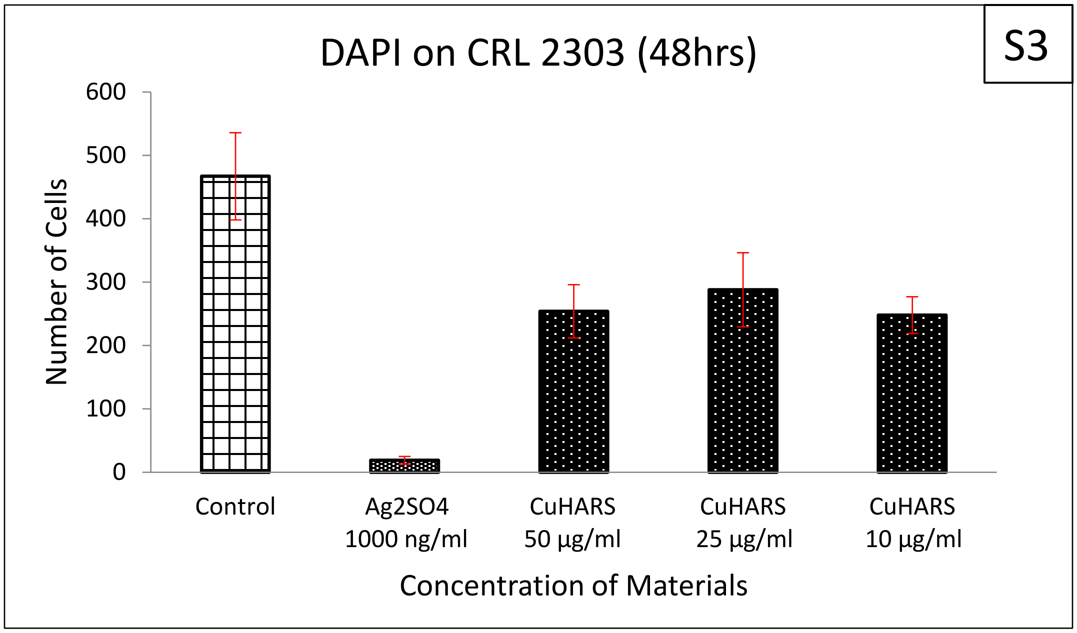

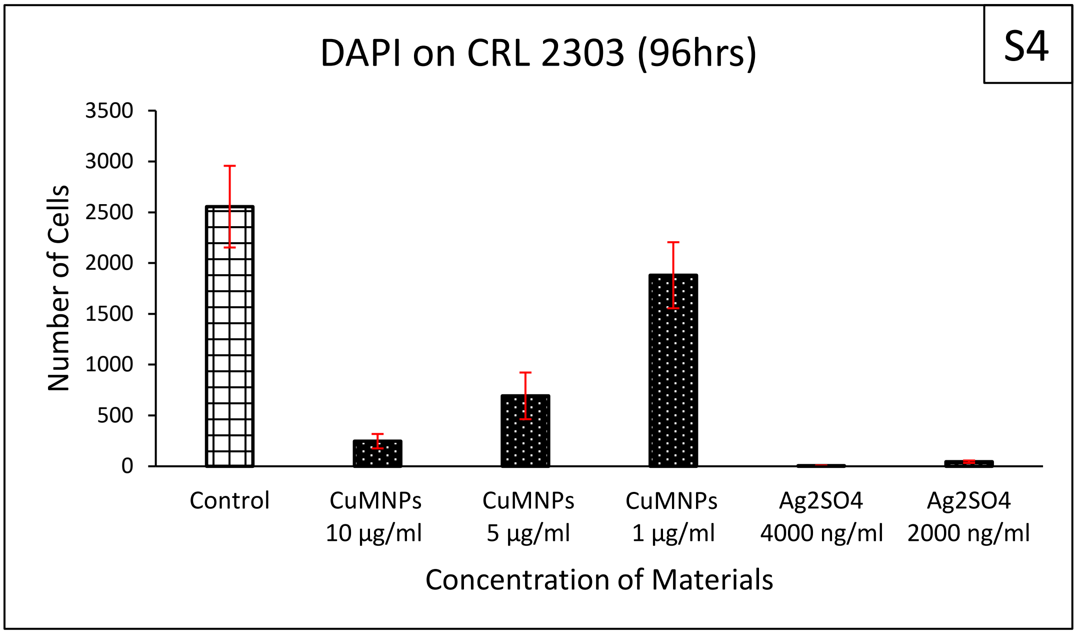

Supporting figures S1-S4: DAPI staining of cell nuclei was carried out as described in methods, and used to assess toxicity of indicated materials on previously plated cells (CRL-2303 glioma), for the time indicated. Image Pro Plus image analysis software (version 7.0) was used to count the number of nuclei (number of cells), as indicated on the Y-axis. For each experiment (S1-S4), data shown are the average of at least 3 fields from 2 separate wells (n=6 total or greater), with standard deviation indicated. Experiments represent multiple microwell plates and multiple platings of cells.
